# Central corneal thickness in newly diagnosed glaucoma patients in South West Ethiopia: a cross-sectional study

**DOI:** 10.1186/s12886-016-0334-x

**Published:** 2016-08-31

**Authors:** Kumale Tolesa, Girum W. Gessesse

**Affiliations:** Jimma University, Ethiopia, P.O. Box: 378, Jimma, Ethiopia

**Keywords:** Central corneal thickness, Intraocular pressure, Pseudoexfoliation, Glaucoma, Ethiopia

## Abstract

**Background:**

Reports of central corneal thickness (CCT) among glaucoma patients, particularly for pseudoexfoliative glaucoma (PXG) and Primary Angle Closure Glaucoma (PACG) are scarce in the Sub-Saharan African (SSA) population. The aim of this study is to evaluate CCT in black patients with newly diagnosed glaucoma and ocular hypertension (OHT) in South West Ethiopia.

**Methods:**

This was a prospective study undertaken with an ultrasonic pachymeter from June 2014 to February 2015 in Jimma University Specialized Hospital. Patients aged 18 years and older newly diagnosed with glaucoma or OHT were included.

**Results:**

A total of 162 eyes of 162 subjects were included. Hundred and fifty five subjects were glaucomatous: (67 PXG, 42 Primary Open Angle Glaucoma (POAG), 28 PACG, 14 Normal Tension Glaucoma (NTG), 5 Juvenile Open Angle Glaucoma (JOAG) and 6 (OHT). The mean age was 59.3 ± 12.8 years.

For the whole sample, CCT was 518.67 (±39.97) μm. OHT group had significantly greater CCT (576.33 ± 49.32 μm) than the glaucomatous groups (*p* = 0.004). POAG (506.69 ± 35.08 μm) and NTG (510.79 ± 44.37 μm) groups had thinner CCT than PXG (520.48 ± 38.95 μm), PACG (524.00 ± 37.16 μm), and JOAG (518.00 ± 30.82 μm) groups, but this was not statistically significant (*p* = 0. 296). There was a statistically significant decline of CCT with advanced age (*P* = 0.02). There wasn’t significant difference of average CCT between the ethnic groups (*P* = 0.3) and gender (*P* = 0.064).

**Conclusion:**

The mean CCT of Ethiopian glaucoma patients is thinner than Caucasians and similar to those reported from previous studies in Sub Saharan Africa. OHT patients had thicker CCT; there was no statistically significant difference observed in average CCT amongst glaucoma subtypes.

## Background

The role of central corneal thickness (CCT) measurement in the clinical evaluation of glaucoma is well established [1–4]. CCT is believed to influence the intraocular pressure (IOP) measured through the cornea with an overestimation in thicker corneas and an underestimation in thinner ones. There are also suggestions of the influence of CCT that is not tonometry-related [5], whereby having thin CCT is associated with development and progression of glaucoma [6–8].

Corneal thickness is among the most highly heritable aspects of ocular structures, suggesting that the gene(s) controlling this ocular structure may vary among populations [9]. Evaluation of measurements from various ethnic groups has provided strong evidence that ethnicity influences CCT [10]. Corneas of black Africans and African Americans are reported to be thinner than that of Caucasians [11–13], while patients with ocular hypertension (OHT) generally have thicker corneas than normals. There is conflicting report in the literature on the differences of CCT among different glaucoma sub-types.

There is paucity of data regarding variations in CCT in different types of glaucoma, particularly in the black population of Sub Saharan Africa (SSA). Primary Open Angle Glaucoma (POAG) is said to be the most common type of glaucoma in this region. Reports of CCT among pseudoexfoliative glaucoma (PXG) (the most common type of secondary open-angle glaucoma) [14] and primary angle closure glaucoma (PACG) are particularly scarce. Therefore there is a need for studies on CCT values among glaucoma patients in a given clinic, especially in SSA. The aim of this study is to evaluate CCT in black patients newly diagnosed with OHT and glaucoma in South West Ethiopia.

## Methods

This prospective study was conducted in the glaucoma service of a tertiary hospital in South West Ethiopia from June 2014 to February 2015. Verbal informed consent was obtained from all subjects who participated in this study.

### Inclusion criterion

All patients aged 18 years and above and newly diagnosed to have glaucoma or OHT were included.

### Exclusion criteria

Eyes with corneal disease or surgery, previous intraocular surgery (particularly cataract and/or glaucoma surgeries), and patients already on anti-glaucoma medications were excluded from the study.

### Procedure

All patients had comprehensive eye examination consisting of an evaluation of visual acuity on the Snellen chart, a biomicroscopic evaluation of the anterior segment with slit lamp biomicroscopy (Zeiss model), fundus examination with a 90 diopter Volk lens, IOP measured using Goldman applanation tonometry and gonioscopy done using Zeiss 4mirror lens. CCT was measured by a contact ultrasound pachymeter (Pachmate DGH55, DGH-KOI, Inc. Shermans Dale, PA, USA). After application of local anesthetic agent, CCT was measured from the center of the pupils holding the probe perpendicular to the cornea, in forward sight and sitting position of patient. An average of twenty-five consecutive measurements were recorded in each eye, such that each recording had a standard deviation <5 μm. All measurements were done by the same author (KT).

Data from the right eye was randomly used for analysis. Data from left eye was included if the right eye was normal or had any of the above mentioned exclusion criteria.

### Group definitions

A diagnosis of glaucoma was made based on the presence of characteristic glaucomatous optic neuropathy (GON) with or without visual field changes on Humphrey frequency doubling technology (FDT) using the N-30 program (Zeiss Humphrey Systems, Dublin, CA). The minimum criteria for early glaucomatous visual field defect were: One or more abnormal points in the central 5 areas and in the 2 non-peripheral nasal areas, and/or more than 1 *P* < 5 % defect or at least 1 *P* < 2 % defect in the periphery. GON in this study was defined as the presence of vertical CDR greater than 0.4, associated with notching, vertical elongation of the cup, nerve fiber layer defect or a difference in the vertical CDR of > = 0.2 between the eyes. OHT was defined as untreated IOP > 21 mmHg with no optic nerve head changes suggesting GON and normal VF. POAG was diagnosed on the basis of an examination of an open irido-corneal angle of 360°, no angle pathology, with IOP 22 mmHg or above in adults above 35 years of age, while JOAG was diagnosed in those under 35 years of age yet meeting the other criteria of POAG. NTG was diagnosed if the IOP was < 22 mmHg with open angles. PACG was defined in association with a closed angle (presence of at least 180° of angle in which the posterior trabecular meshwork was not visible on non-indentation gonioscopy), and IOP more than 21 mmHg. All PACG patients included had chronic PACG. Diagnosis of PXG was based on the presence of characteristic exfoliated material on the pupil margin or anterior surface of the lens on biomicroscopy in addition to 1 or more of the following findings on gonioscopy: heavy angle pigmentation, Sampaolesi line or exfoliated material in the angle recess.

### Statistical analysis

Statistical analysis was performed with SPSS (version 16. for Windows XP; SPSS Inc., Chicago, IL, USA). Simple linear regression analysis was used to examine association between CCT and continuous variables such as age. A one way analysis of variance (ANOVA) and Student’s *t*-test were used to test for differences in CCT between glaucoma groups. Logistic analyses were used to assess any relationship between CCT and glaucoma type after accounting for age, sex, and IOP at diagnosis. A P value of less than 0.05 was considered statistically significant.

## Results

### Population characteristics

A total of 162 eyes of 162 consecutive patients were included in this study. All recruited patients were black Ethiopian citizens. They comprised 108 males (66.7 %) and 54 women (33.3 %). Average age of the study population was 59.3 ± 12.8 years (ranging from 20 to 100 years). The average age of females was 55.4 ± 11.8 years while that of men was 61.2 ± 12.8 years. This difference was statistically significant (*p* = 0.006; *t* test).

PXG was the most common clinical diagnosis in 67 patients (41.4 %), followed by POAG, 42 (25.9 %), and PACG, 28 (17.3 %), as per Table 1. Average age (±SD) in the PXG, POAG, PACG, NTG and OHT groups was 64.6 (±10.8), 60.4 (±10.3), 53.2 (±9.9), 57.8 (±10.3), and 51.5 (±16.1) years respectively. The differences were statistically significant (*p* < 0.01). JOAG patients had average age of 26.4 ± 6.3 years.

**Table 1:** Characteristics of the population

Demographic characteristics	Number (%)
Age -mean (SD)	59.3 (12.8)
Female	54 (33.3 %)
Male	108 (66.7 %)
Ethnic group	
Cushitic	93 (57.4 %)
Omotic	33 (20.4 %)
Semitic	26 (16.0 %)
Nilo-Saharan	5 (3.1 %)
Mixed	5 (3.1 %)
Clinical Subtypes	
PXG	67 (41.4 %)
POAG	42 (25.9 %)
PACG	28 (17.3 %)
NTG	14 (8.6 %)
OHT	6 (3.7 %)
JOAG	5 (3.1 %)

Abbreviations *PXG* Pseudoexfoliative glaucoma, *POAG* Primary Open Angle Glaucoma, *PACG* Primary Open Angle Closure Glaucoma, *NTG* Normal Tension Glaucoma, *OHT* Ocular Hypertension, *JOAG* Juvenile Open Angle Glaucoma

### Intraocular pressure

Average IOP of the study population was 33.5 ± 10.99 mmHg; Average IOP was not different between males, (33.9 ± 10.7 mmHg) and females (32.9 ± 11.7 mmHg), (*p* = 0.58). The mean IOP for PXG (37.4 ± 9.6 mmHg), PACG (36.0 ± 11.0) and JOAG (38.2 ± 13.1) patients were higher than those with POAG patients (31.4 ± 9.5) and OHT (28.8 ± 3.9) patients. This difference was statistically significant (*p* < 0.01, *t* test, Table 2).

**Table 2:** Mean age, intraocular pressure (IOP) and Central corneal thickness (CCT) among the clinical sub- groups

Clinical Subtypes	Mean Age (SD) in years	Mean IOP (SD) mmHg	Mean CCT (SD) μm
PXG	64.6 (10.8)	37.4 (9.6)	520.48 (38.95)
POAG	60.4 (10.3)	31.4 (9.5)	506.69 (35.08)
PACG	53.2 (9.9)	36.0 (11.0)	524.00 (37.16)
NTG	57.8 (10.3)	16.8 (2.8)	510.79 (44.37)
OHT	51.5 (16.1)	28.8 (3.9)	576.33 (49.32)
JOAG	26.4 (6.3)	38.2 (13.1)	518.00 (30.82)
*p*	<0.01	<0.01	0.003

Abbreviations *PXG* Pseudoexfoliative glaucoma, *POAG* Primary Open Angle Glaucoma, *PACG* Primary Open Angle Closure Glaucoma, *NTG* Normal Tension Glaucoma, *OHT* Ocular Hypertension, *JOAG* Juvenile Open Angle Glaucoma

### Central corneal thickness

**Fig. 1:**
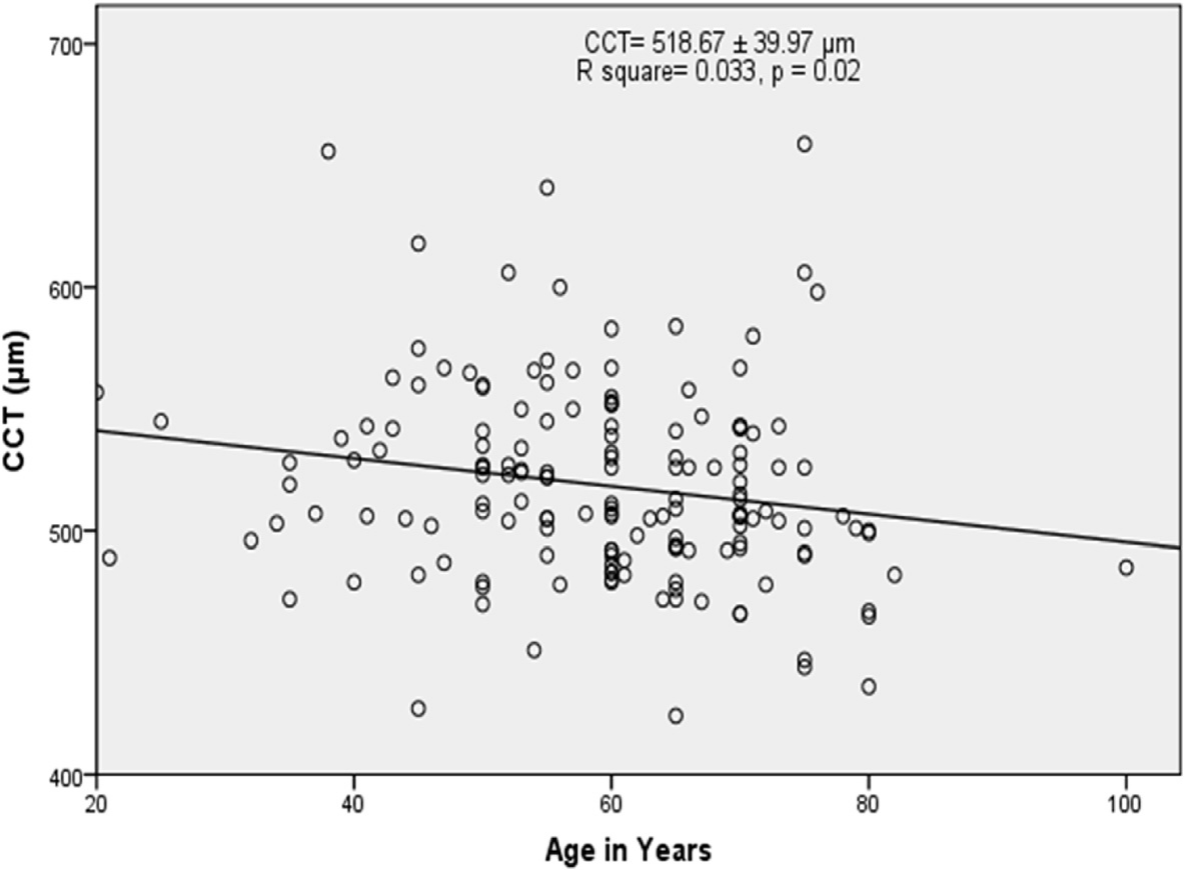
Scattergram of central corneal thickness (CCT) versus age (*n* = 162 eyes)

Average CCT of the study population was 518.67 ± 39.97 μm (min- max: 424–659). Linear regression analysis showed that there was a statistically significant decline of CCT with advanced age (r^2^ = 033, *p* = 0.02, Fig. 1). This trend was found to be similar in males (r^2^ = 0.049, *p* = 0.022), but the decline with age in females was not statistically significant (r^2^ = 0.00, *p* = 0.91).

### Central corneal thickness and gender, ethnic group

Females had an average CCT of 526.89 ± 39.36 μm (min- max: 424–641) while men had an average CCT of 514.56 ± 39.82 μm (min- max: 436–659). The difference was not statistically significant (*P* = 0.064). CCT was also not significantly different between the ethnic groups (*P* = 0.3, Table 3).

**Table 3:** Central corneal thickness (CCT) according to age and ethnicity

Demographic characteristics		Mean CCT (SD)
Age (years)	20– 39	528.18 (49.44)
	40 – 54	525.54 (38.69)
	55 – 64	521.96 (36.91)
	65 – 74	512.26 (31.37)
	75+	505.72 (59.08)
CEthnicity	Cushitic	518.71 (37.90)
	Omotic	522.67 (37.42)
	Semitic	520.50 (52.26)
	Nilo-Saharan	480.40 (6.23)
	Mixed	520.20 (31.25)

### Central corneal thickness according to group

Average (±SD) CCT in the subtypes were: PXG (520.48 ± 38.95 μm), POAG (506.69 ± 35.08 μm), PACG (524.00 ± 37.16 μm), NTG (510.79 ± 44.37 μm), JOAG (518.00 ± 30.82 μm), and OHT (576.33 ± 49.32 μm) patients. This shows OHT patients had significantly thicker CCT than the glaucoma groups (*p* = 0.004, *t* test). The linear regression shows that this difference in CCT between the groups was not correlated with age or gender. Excluding OHT patients, POAG (506.69 ± 35.08 μm) and NTG (510.79 ± 44.37 μm) groups had slightly thinner average CCT than PXG, PACG, and JOAG groups. However, the difference among the glaucoma subtypes was not statistically significant (*p* = 0.296, *t* test).

With regression analysis, no linear correlation was observed between CCT and IOP (*r* = 0.112, *p* = 0.16).

## Discussion

While CCT affects IOP measurement by GAT, the relationship between CCT and either glaucoma risk or glaucoma progression cannot be explained exclusively by tonometry artifact. Reports show thin CCT to be the strongest predictor of development of glaucoma among ocular hypertensive patients. Glaucoma patients with thin CCT tend to have a worse outcome, including more severe glaucomatous damage at presentation, and higher risk of disease progression [5–8]. It is therefore very important to measure CCT in patients as part of a glaucoma workup. Studying CCT in a given clinic also helps in better understanding the variation of this parameter among glaucoma subtypes and different racial/ethnic groups.

PXG and PACG are considered infrequent subtypes in SSA. In our clinical series, PXG was the most common type (41.4 %) and PACG accounted for 17.3 % of the cases; this is in agreement with a previous clinic based report of glaucoma subtypes in the same hospital [15]. PXG patients tended to present at older age (64.6 ± 10.8 years) while PACG (53.2 ± 9.9 years) and OHT (51.5 ± 16.1 years) patients were younger. This was statistically significant (*p* < 0.01). PXG eyes also presented with higher IOP (37.4 ± 9.6 mmHg) than the other groups. These findings support the evidence that PXG patients present at older age, with higher baseline IOPs than other types, particularly POAG [16].

The mean CCT of glaucoma patients in this study was 518.67 (±39.97) μm. This is in agreement with the existing evidence that black Africans have generally thinner CCT than whites and other races [12, 13]. Similar findings have been reported from clinic-based studies among black glaucoma patients in Ethiopia (518.68 ± 32.92 μm) [17], Uganda (516.19 ± 39.95 μm); [18] and African Americans (518 μm) [19]. Among patients with glaucoma in West Africa, findings ranged from 524.28 μm to 536.91 μm [12, 20, 21].

Reports on the association between CCT and age are inconsistent. Our study showed a statistically significant inverse relationship, with decreasing of CCT with advancing age. This is in agreement with other studies from Africa [12, 22, 23]. However, the decline of CCT with age in females was not statistically significant (r^2^ = 0.00, *p* = 0.91). This may be because this study had a smaller sample size of females and, they had a younger average age at presentation. The weak association between CCT and IOP observed in our study is also consistent with that of other population-based studies [11, 12, 24, 25].

Average CCT was significantly greater in OHT patients (576.33 ± 49.32) than the glaucomatous groups. This is in agreement with earlier reports. The average value was, however greater than those reported from other studies in Ethiopia (524.32 μm) and Cameroon (547.32 ± 35.7 μm) [21]. Although average CCT were relatively thinner among POAG (506.69 ± 35.08) and NTG (510.79 ± 44.37) patients, the differences observed with the other glaucoma subtypes was not statistically significant (*p* = 0.296, *t* test). This finding among POAG is one of the lowest CCT values reported from across the globe. Similar findings of average CCT have been reported among POAG subgroups in Ethiopia (502.24 μm) [17] and Black South Africans (506.0 μm) [26], while reports from other SSA region range from 519.6 ± 32.6 μm-526.30 ± 37.34 μm [20, 21, 27].

We didn’t find adequate published data from the SSA region on CCT among PXG and PACG patients for comparison with our results. There are conflicting reports on the differences of mean CCT between patients with PXG and other diagnostic groups, particularly POAG. Similar to some reports from Europe [25, 28], we found no statistically significant difference of CCT between PXG and POAG subjects. Some studies from other regions have shown PXG eyes to have thinner CCT compared to POAG and normal eyes [19, 29, 30], while others found thicker CCT in PXG compared to POAG eyes. Although our PXG patients had older mean age (which was associated with thinner CCT) and higher IOP, these were not found to affect the overall CCT on regression analysis. A study from central Ethiopia reported CCT in PXG eyes of 579.00 μm [17]. However, there were only 4 PXG eyes included in the study, with surgery also a possibility to have affected the results. More studies from different regions, with comparison of CCT in subsets of patients with pseudoexfoliative syndrome (with and without glaucoma), POAG and normal controls would better provide more definitive information. With regard to PACG, our study is also in agreement with reports from Asia that showed that PACG eyes had similar CCT to those with POAG [31, 32].

### Limitations of the study

A major limitation of this study is the clinic-based setting of our patient selection as well as the small number of patients in OHT and NTG groups. However, the prospective nature of the study, with all baseline measurement of IOP and CCT taken at the initial diagnosis, no medication or surgical intervention to possibly affect the measurements, are the strengths. It is also the first study in the region to describe CCT among a large sample of patients with PXG and PACG, which are considered to be uncommon in SSA.

## Conclusion

The mean CCT of Ethiopian glaucoma patients in the region is thinner than whites and similar to those reported from SSA. OHT patients had thicker CCT; Thinner CCT was found among POAG and NTG patients, while those of PACG and PXG had intermediate. However, there was no statistically significant difference observed in average CCT between glaucoma subtypes.

## Abbreviations

CCT: Central corneal thickness
CDR: Cup- disc ratio
GAT: Goldman applanation tonometry
GON: Glaucomatous optic neuropathy
IOP: Intraocular pressure
JOAG: Juvenile open angle glaucoma
NTG: Normal tension glaucoma
OHT: Ocular hypertension
PACG: Primary angle closure glaucoma
POAG: Primary open angle glaucoma
PXG: Pseudoexfoliative glaucoma
SSA: Sub- Saharan Africa
